# *catena*-Poly[[(4-amino­benzoato)aqua­silver(I)]-μ-hexa­methyl­enetetramine]

**DOI:** 10.1107/S1600536809055044

**Published:** 2010-01-09

**Authors:** Jia-Jun Han, Tian-Yun Zhang, Lin Geng

**Affiliations:** a School of the Ocean, Harbin Institute of Technology, Weihai 264209, People’s Republic of China; b School of Materials Science and Engineering, Harbin Institute of Technology, Harbin 150001, People’s Republic of China

## Abstract

In the title coordination polymer, [Ag(C_7_H_6_NO_2_)(C_6_H_12_N_4_)(H_2_O)]_*n*_, the Ag^I^ ion is five-coordinated by two carboxyl­ate O atoms from one 4-amino­benzoate anion (*L*), two N atoms from two different hexa­methyl­enetetramine (hmt) ligands, and one water O atom in a distorted square-pyramidal geometry. The metal atom lies on a mirror plane and the *L* anion, hmt ligand and water mol­ecule all lie across crystallographic mirror planes. Each hmt ligand bridges two neighboring Ag^I^ ions, resulting in the formation of a chain structure along the *b* axis. The chains are linked into a three-dimensional framework by N—H⋯O and O—H⋯O hydrogen bonds.

## Related literature

For the applications and structures of silver(I) coordination polymers, see: Yang *et al.* (2007 ▶, 2008 ▶). 
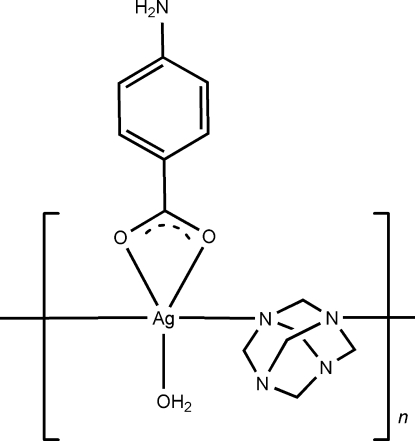


## Experimental

### 

#### Crystal data

[Ag(C_7_H_6_NO_2_)(C_6_H_12_N_4_)(H_2_O)]
*M* *_r_* = 402.21
Orthorhombic, 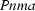

*a* = 19.8107 (11) Å
*b* = 6.4877 (3) Å
*c* = 11.3257 (6) Å
*V* = 1455.65 (13) Å^3^
*Z* = 4
Mo *K*α radiation
μ = 1.41 mm^−1^
*T* = 293 K
0.31 × 0.27 × 0.22 mm

#### Data collection

Bruker APEX CCD area-detector diffractometer
Absorption correction: multi-scan (*SADABS*; Sheldrick, 1996 ▶) *T*_min_ = 0.66, *T*_max_ = 0.87
7757 measured reflections
1557 independent reflections
1373 reflections with *I* > 2σ(*I*)
*R*_int_ = 0.029

#### Refinement

*R*[*F* ^2^ > 2σ(*F* ^2^)] = 0.019
*wR*(*F* ^2^) = 0.048
*S* = 1.08
1557 reflections
123 parameters
H atoms treated by a mixture of independent and constrained refinement
Δρ_max_ = 0.37 e Å^−3^
Δρ_min_ = −0.26 e Å^−3^

### 

Data collection: *SMART* (Bruker, 1998 ▶); cell refinement: *SAINT* (Bruker, 1998 ▶); data reduction: *SAINT*; program(s) used to solve structure: *SHELXS97* (Sheldrick, 2008 ▶); program(s) used to refine structure: *SHELXL97* (Sheldrick, 2008 ▶); molecular graphics: *SHELXTL* (Sheldrick, 2008 ▶); software used to prepare material for publication: *SHELXTL*.

## Supplementary Material

Crystal structure: contains datablocks global, I. DOI: 10.1107/S1600536809055044/ci2988sup1.cif

Structure factors: contains datablocks I. DOI: 10.1107/S1600536809055044/ci2988Isup2.hkl

Additional supplementary materials:  crystallographic information; 3D view; checkCIF report

## Figures and Tables

**Table 1:** Selected bond lengths (Å)

Ag1—N1	2.3862 (17)
Ag1—O1*W*	2.445 (2)
Ag1—O1	2.5413 (14)

**Table 2:** Hydrogen-bond geometry (Å, °)

*D*—H⋯*A*	*D*—H	H⋯*A*	*D*⋯*A*	*D*—H⋯*A*
O1*W*—H1*W*1⋯O1^i^	0.81 (2)	1.95 (2)	2.742 (2)	168 (2)
N2—H2*A*⋯O1^ii^	0.84 (2)	2.27 (2)	3.072 (2)	159 (2)

Symmetry codes: (i) 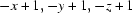; (ii) 
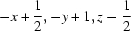
.

**Fig. 1.**
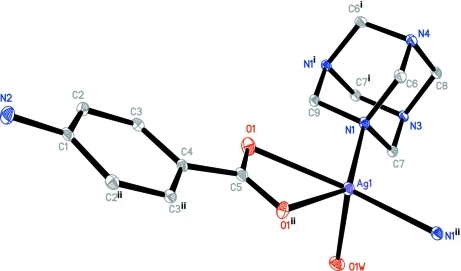
A view of the local coordination of the AgI centre in the title compound, showing the atom-labelling scheme. Displacement ellipsoids are drawn at the 30% probability level. Symmetry codes: (i) x, 3/2 - y, z; (ii) x, 1/2 - y, z.

**Fig. 2.**
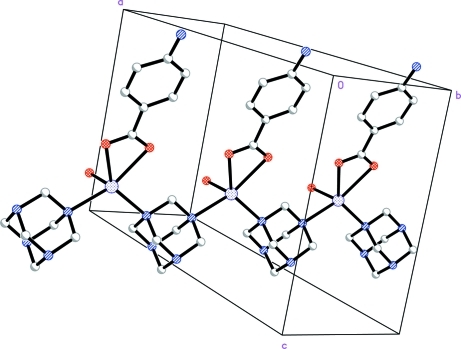
Part of the polymeric chain in the title compound.

## supplementary crystallographic information

### Experimental

An aqueous solution (10 ml) of HL (0.104 g, 0.5 mmol) was added to solid Ag_2_CO_3_ (0.25 mmol) and stirred for several minutes until no further CO_2_ was given off. A solution of hmt (0.5 mmol) in acetonitrile (10 ml) was then added and a white precipitate formed. The precipitate was dissolved by dropwise addition of an aqueous solution of NH_3_ (14 M). Colourless blocks of the title compound were obtained by evaporation of the solution for several days at room temperature (33% yield).

### Refinement

Amino and water H-atoms were located in a difference Fourier map, and refined freely. The remaining H atoms were positioned geometrically (C-H = 0.93 Å) and refined as riding, with *U*_iso_(H) = 1.2*U*_eq_(C).

### Crystal data

**Table:**

[Ag(C_7_H_6_NO_2_)(C_6_H_12_N_4_)(H_2_O)]	*F*(000) = 816
*M**_r_* = 402.21	*D*_x_ = 1.835 Mg m^−^^3^
Orthorhombic, *P**n**m**a*	Mo *K*α radiation, λ = 0.71073 Å
Hall symbol: -P 2ac 2n	Cell parameters from 1557 reflections
*a* = 19.8107 (11) Å	θ = 3.0–26.0°
*b* = 6.4877 (3) Å	µ = 1.41 mm^−^^1^
*c* = 11.3257 (6) Å	*T* = 293 K
*V* = 1455.65 (13) Å^3^	Block, colourless
*Z* = 4	0.31 × 0.27 × 0.22 mm

### Data collection

**Table:**

Bruker APEX CCD area-detector diffractometer	1557 independent reflections
Radiation source: fine-focus sealed tube	1373 reflections with *I* > 2σ(*I*)
graphite	*R*_int_ = 0.029
φ and ω scans	θ_max_ = 26.0°, θ_min_ = 2.1°
Absorption correction: multi-scan (*SADABS*; Sheldrick, 1996)	*h* = −24→23
*T*_min_ = 0.66, *T*_max_ = 0.87	*k* = −7→7
7757 measured reflections	*l* = −13→9

### Refinement

**Table:**

Refinement on *F*^2^	Primary atom site location: structure-invariant direct methods
Least-squares matrix: full	Secondary atom site location: difference Fourier map
*R*[*F*^2^ > 2σ(*F*^2^)] = 0.019	Hydrogen site location: inferred from neighbouring sites
*wR*(*F*^2^) = 0.048	H atoms treated by a mixture of independent and constrained refinement
*S* = 1.08	*w* = 1/[σ^2^(*F*_o_^2^) + (0.0211*P*)^2^ + 0.5726*P*] where *P* = (*F*_o_^2^ + 2*F*_c_^2^)/3
1557 reflections	(Δ/σ)_max_ = 0.001
123 parameters	Δρ_max_ = 0.37 e Å^−^^3^
0 restraints	Δρ_min_ = −0.26 e Å^−^^3^

### Special details

**Table:**

Geometry. All esds (except the esd in the dihedral angle between two l.s. planes) are estimated using the full covariance matrix. The cell esds are taken into account individually in the estimation of esds in distances, angles and torsion angles; correlations between esds in cell parameters are only used when they are defined by crystal symmetry. An approximate (isotropic) treatment of cell esds is used for estimating esds involving l.s. planes.
Refinement. Refinement of F^2^ against ALL reflections. The weighted R-factor wR and goodness of fit S are based on F^2^, conventional R-factors R are based on F, with F set to zero for negative F^2^. The threshold expression of F^2^ > 2sigma(F^2^) is used only for calculating R-factors(gt) etc. and is not relevant to the choice of reflections for refinement. R-factors based on F^2^ are statistically about twice as large as those based on F, and R- factors based on ALL data will be even larger.

### Fractional atomic coordinates and isotropic or equivalent isotropic displacement parameters (Å^2^)

**Table:**

	*x*	*y*	*z*	*U*_iso_*/*U*_eq_	Occ. (<1)
Ag1	0.425226 (10)	0.2500	0.638361 (18)	0.02218 (9)	
O1W	0.54862 (12)	0.2500	0.6362 (2)	0.0324 (5)	
H1W1	0.5643 (11)	0.352 (4)	0.606 (2)	0.036 (7)*	
O1	0.38093 (7)	0.4203 (2)	0.45173 (12)	0.0306 (4)	
N1	0.42059 (7)	0.5604 (3)	0.75114 (14)	0.0198 (4)	
N2	0.23019 (13)	0.2500	−0.0371 (2)	0.0261 (6)	
H2A	0.2075 (11)	0.358 (3)	−0.0497 (19)	0.033 (7)*	
N3	0.47738 (11)	0.7500	0.9101 (2)	0.0186 (5)	
N4	0.35383 (11)	0.7500	0.8979 (2)	0.0228 (5)	
C1	0.26785 (13)	0.2500	0.0657 (2)	0.0182 (6)	
C2	0.28812 (9)	0.4347 (3)	0.11800 (16)	0.0206 (4)	
H2	0.2781	0.5594	0.0814	0.025*	
C3	0.32294 (9)	0.4339 (3)	0.22365 (16)	0.0196 (4)	
H3	0.3357	0.5585	0.2576	0.024*	
C4	0.33924 (13)	0.2500	0.2803 (2)	0.0178 (6)	
C5	0.36952 (13)	0.2500	0.4018 (2)	0.0222 (6)	
C6	0.35759 (9)	0.5670 (3)	0.82288 (17)	0.0235 (5)	
H6A	0.3189	0.5649	0.7704	0.028*	
H6B	0.3554	0.4448	0.8721	0.028*	
C7	0.47854 (9)	0.5664 (3)	0.83436 (16)	0.0214 (4)	
H7A	0.4776	0.4440	0.8836	0.026*	
H7B	0.5203	0.5647	0.7897	0.026*	
C8	0.41317 (12)	0.7500	0.9761 (2)	0.0223 (6)	
H8A	0.4115	0.6292	1.0264	0.027*	0.50
H8B	0.4115	0.8708	1.0264	0.027*	0.50
C9	0.42303 (13)	0.7500	0.6783 (2)	0.0200 (6)	
H9A	0.4642	0.7500	0.6321	0.024*	
H9B	0.3852	0.7500	0.6240	0.024*	

### Atomic displacement parameters (Å^2^)

**Table:**

	*U*^11^	*U*^22^	*U*^33^	*U*^12^	*U*^13^	*U*^23^
Ag1	0.02328 (13)	0.02679 (14)	0.01649 (13)	0.000	−0.00302 (9)	0.000
O1W	0.0240 (12)	0.0378 (15)	0.0352 (14)	0.000	0.0073 (10)	0.000
O1	0.0353 (8)	0.0378 (9)	0.0187 (7)	−0.0053 (8)	−0.0049 (6)	−0.0054 (7)
N1	0.0170 (8)	0.0292 (10)	0.0130 (8)	−0.0014 (7)	−0.0024 (6)	0.0004 (7)
N2	0.0244 (14)	0.0349 (17)	0.0189 (13)	0.000	−0.0072 (11)	0.000
N3	0.0137 (11)	0.0288 (14)	0.0134 (11)	0.000	0.0003 (9)	0.000
N4	0.0140 (11)	0.0378 (15)	0.0165 (12)	0.000	−0.0014 (9)	0.000
C1	0.0126 (12)	0.0283 (15)	0.0137 (14)	0.000	0.0039 (11)	0.000
C2	0.0208 (10)	0.0229 (11)	0.0179 (10)	0.0016 (8)	0.0010 (8)	0.0035 (8)
C3	0.0200 (10)	0.0210 (11)	0.0179 (10)	−0.0011 (8)	0.0041 (8)	−0.0020 (8)
C4	0.0112 (12)	0.0279 (16)	0.0143 (13)	0.000	0.0024 (10)	0.000
C5	0.0145 (13)	0.0355 (18)	0.0168 (14)	0.000	0.0022 (11)	0.000
C6	0.0159 (9)	0.0355 (13)	0.0192 (10)	−0.0040 (9)	−0.0024 (8)	0.0019 (9)
C7	0.0167 (10)	0.0308 (12)	0.0167 (10)	0.0015 (9)	−0.0025 (8)	0.0019 (8)
C8	0.0152 (14)	0.0377 (18)	0.0139 (14)	0.000	−0.0018 (11)	0.000
C9	0.0168 (13)	0.0301 (17)	0.0132 (13)	0.000	−0.0041 (11)	0.000

### Geometric parameters (Å, °)

**Table:**

Ag1—N1	2.3862 (17)	C1—C2	1.396 (2)
Ag1—N1^i^	2.3862 (17)	C1—C2^i^	1.396 (2)
Ag1—O1W	2.445 (2)	C2—C3	1.381 (3)
Ag1—O1	2.5413 (14)	C2—H2	0.93
Ag1—O1^i^	2.5413 (14)	C3—C4	1.393 (2)
O1W—H1W1	0.81 (2)	C3—H3	0.93
O1—C5	1.2615 (19)	C4—C3^i^	1.393 (2)
N1—C9	1.482 (2)	C4—C5	1.500 (4)
N1—C7	1.486 (2)	C5—O1^i^	1.2615 (19)
N1—C6	1.490 (2)	C6—H6A	0.97
N2—C1	1.383 (4)	C6—H6B	0.97
N2—H2A	0.84 (2)	C7—H7A	0.97
N3—C7	1.468 (2)	C7—H7B	0.97
N3—C7^ii^	1.468 (2)	C8—H8A	0.97
N3—C8	1.475 (3)	C8—H8B	0.97
N4—C6	1.462 (2)	C9—N1^ii^	1.482 (2)
N4—C6^ii^	1.462 (2)	C9—H9A	0.97
N4—C8	1.472 (3)	C9—H9B	0.97
			
N1—Ag1—N1^i^	115.09 (8)	C2—C3—H3	119.4
N1—Ag1—O1W	92.52 (5)	C4—C3—H3	119.4
N1^i^—Ag1—O1W	92.52 (5)	C3^i^—C4—C3	117.9 (2)
N1—Ag1—O1	93.74 (5)	C3^i^—C4—C5	121.01 (12)
N1^i^—Ag1—O1	143.00 (5)	C3—C4—C5	121.01 (12)
O1W—Ag1—O1	109.69 (6)	O1^i^—C5—O1	122.2 (3)
N1—Ag1—O1^i^	143.00 (5)	O1^i^—C5—C4	118.87 (13)
N1^i^—Ag1—O1^i^	93.74 (5)	O1—C5—C4	118.87 (13)
O1W—Ag1—O1^i^	109.69 (6)	N4—C6—N1	112.52 (17)
O1—Ag1—O1^i^	51.53 (7)	N4—C6—H6A	109.1
Ag1—O1W—H1W1	112.9 (17)	N1—C6—H6A	109.1
C5—O1—Ag1	93.12 (14)	N4—C6—H6B	109.1
C9—N1—C7	107.80 (16)	N1—C6—H6B	109.1
C9—N1—C6	107.87 (17)	H6A—C6—H6B	107.8
C7—N1—C6	107.49 (15)	N3—C7—N1	112.34 (17)
C9—N1—Ag1	113.67 (12)	N3—C7—H7A	109.1
C7—N1—Ag1	109.39 (12)	N1—C7—H7A	109.1
C6—N1—Ag1	110.39 (12)	N3—C7—H7B	109.1
C1—N2—H2A	115.4 (16)	N1—C7—H7B	109.1
C7—N3—C7^ii^	108.4 (2)	H7A—C7—H7B	107.9
C7—N3—C8	108.02 (14)	N4—C8—N3	112.6 (2)
C7^ii^—N3—C8	108.02 (14)	N4—C8—H8A	109.1
C6—N4—C6^ii^	108.6 (2)	N3—C8—H8A	109.1
C6—N4—C8	107.99 (14)	N4—C8—H8B	109.1
C6^ii^—N4—C8	107.99 (14)	N3—C8—H8B	109.1
N2—C1—C2	120.83 (12)	H8A—C8—H8B	107.8
N2—C1—C2^i^	120.83 (12)	N1^ii^—C9—N1	112.3 (2)
C2—C1—C2^i^	118.3 (2)	N1^ii^—C9—H9A	109.2
C3—C2—C1	120.53 (19)	N1—C9—H9A	109.2
C3—C2—H2	119.7	N1^ii^—C9—H9B	109.2
C1—C2—H2	119.7	N1—C9—H9B	109.2
C2—C3—C4	121.23 (19)	H9A—C9—H9B	107.9
			
N1—Ag1—O1—C5	−166.41 (14)	Ag1—O1—C5—C4	178.7 (2)
N1^i^—Ag1—O1—C5	−23.97 (18)	C3^i^—C4—C5—O1^i^	0.9 (4)
O1W—Ag1—O1—C5	99.57 (14)	C3—C4—C5—O1^i^	177.1 (2)
O1^i^—Ag1—O1—C5	−0.38 (15)	C3^i^—C4—C5—O1	−177.1 (2)
N1^i^—Ag1—N1—C9	−179.54 (11)	C3—C4—C5—O1	−0.9 (4)
O1W—Ag1—N1—C9	86.50 (15)	C6^ii^—N4—C6—N1	58.4 (3)
O1—Ag1—N1—C9	−23.43 (14)	C8—N4—C6—N1	−58.5 (2)
O1^i^—Ag1—N1—C9	−41.73 (17)	C9—N1—C6—N4	−57.9 (2)
N1^i^—Ag1—N1—C7	59.91 (14)	C7—N1—C6—N4	58.1 (2)
O1W—Ag1—N1—C7	−34.05 (13)	Ag1—N1—C6—N4	177.33 (13)
O1—Ag1—N1—C7	−143.98 (12)	C7^ii^—N3—C7—N1	−58.6 (2)
O1^i^—Ag1—N1—C7	−162.28 (10)	C8—N3—C7—N1	58.2 (2)
N1^i^—Ag1—N1—C6	−58.17 (14)	C9—N1—C7—N3	58.2 (2)
O1W—Ag1—N1—C6	−152.14 (12)	C6—N1—C7—N3	−57.8 (2)
O1—Ag1—N1—C6	97.94 (12)	Ag1—N1—C7—N3	−177.73 (13)
O1^i^—Ag1—N1—C6	79.64 (15)	C6—N4—C8—N3	58.64 (14)
N2—C1—C2—C3	176.9 (2)	C6^ii^—N4—C8—N3	−58.64 (14)
C2^i^—C1—C2—C3	−4.8 (4)	C7—N3—C8—N4	−58.54 (13)
C1—C2—C3—C4	0.5 (3)	C7^ii^—N3—C8—N4	58.54 (13)
C2—C3—C4—C3^i^	3.8 (4)	C7—N1—C9—N1^ii^	−58.1 (2)
C2—C3—C4—C5	−172.5 (2)	C6—N1—C9—N1^ii^	57.7 (2)
Ag1—O1—C5—O1^i^	0.7 (3)	Ag1—N1—C9—N1^ii^	−179.56 (11)

Symmetry codes: (i) *x*, −*y*+1/2, *z*; (ii) *x*, −*y*+3/2, *z*.

### Hydrogen-bond geometry (Å, °)

**Table:**

*D*—H···*A*	*D*—H	H···*A*	*D*···*A*	*D*—H···*A*
O1W—H1W1···O1^iii^	0.81 (2)	1.95 (2)	2.742 (2)	168 (2)
N2—H2A···O1^iv^	0.84 (2)	2.27 (2)	3.072 (2)	159 (2)

Symmetry codes: (iii) −*x*+1, −*y*+1, −*z*+1; (iv) −*x*+1/2, −*y*+1, *z*−1/2.
